# The *BNA* Festive Symposium 2025 — delivering neuroscience: From synapse to society

**DOI:** 10.1177/23982128261427975

**Published:** 2026-04-02

**Authors:** Dana Abdulhafiz, Adeyinka Ruth Adeniran, Oluwatobi Ajewole, Limou Dembele, Constantinos Panayiotou, Karina Maria Piotrowska, Tijana Urosevic, Wanjia Yu, Kate Baker

**Affiliations:** 1 IoPPN King’s College London, London, UK; 2 University of Exeter, Exeter, UK; 3 King’s College London, London, UK; 4 Independent researcher, London, UK; 5 Department of Physiology, Anatomy and Genetics, University of Oxford, Oxford, UK; 6 University of Edinburgh, Edinburgh, UK; 7 MRC Cognition and Brain Sciences Unit, University of Cambridge, Cambridge, UK

**Keywords:** Neurological conditions, therapies, neuroscience

## Abstract

The *BNA*’s winter research meeting took place high above the City of London at Canary Wharf, an inspiring setting to consider new perspectives. The central theme was *‘delivery’*, focusing on translation of fundamental neuroscience into effective therapies. The event targeted three main questions: How can therapies be effectively delivered to the brain? How might ‘meaningful intervention’ be realised for patients? How might hope be maintained for people with neurological and psychiatric disorders? Several interlocking ideas and debates emerged, circling around a central concern of how to move from molecular promise to effective and equitable real-world change for patients.

## Session 1: *Delivering Genes to the Brain*

The first session featured three speakers whose work collectively illustrates how modern neuroscience is moving from mechanistic insight to therapeutic delivery. Professor Chris Shaw (Kings College London and AviadoBio) opened the session with an account of his decades-long efforts to translate the genetics of neurodegeneration into meaningful clinical interventions. Drawing on discoveries from amyotrophic lateral sclerosis (ALS) and frontotemporal dementia (FTD), he described the development of AVB-101, a gene therapy designed to restore progranulin in patients with GRN-related FTD (Lee et al., 2025). Delivered directly to the thalamus to enable broad cortical distribution, the therapy has demonstrated promising preclinical results and is now advancing through dose-finding clinical studies. Professor Shaw’s presentation emphasised both the scientific challenge and the human imperative of delivering therapies to families whose genetic contributions made these breakthroughs possible.

The focus then shifted to the problem of how advanced therapies travel through the body. Dr Paul Sharp from the Medicines Discovery Catapult (https://md.catapult.org.uk/) highlighted the crucial role of preclinical imaging in understanding the biodistribution and pharmacokinetics of complex modalities such as viral vectors and nanoparticles. He outlined how contemporary imaging platforms (MRI, PET/CT and high-resolution optical techniques) can reveal where a therapy goes, how long it persists and whether it reaches its intended neural targets. Dr Sharp positioned imaging not as a passive observational tool but as an active driver of therapeutic design and optimisation.

Closing the session, Professor Simon Ward (Medicines Discovery Institute, Cardiff University) offered a complementary perspective from the world of neuropsychiatric drug discovery. He discussed the need for novel small molecule therapeutics that more precisely modulate neural circuits, particularly those governing cognition, mood and social behaviour. Professor Ward emphasised the importance of translational pathways that link early mechanistic research with scalable, clinically viable compounds, highlighting how academia and industry partnerships are reshaping the neurotherapeutic landscape. Together, the speakers presented a compelling narrative: breakthroughs in genetics, imaging and medicinal chemistry are converging to make previously intractable brain disorders newly approachable.

## Session 2: *Delivering in the Clinic*

The second session brought together cross-sector experts to explore opportunities and challenges in clinical translation. The session opened with the *BNA* Prize Lecture ‘*Developing Genetic Therapies for Huntington’s Disease –Trials and Tribulations’* by Professor Sarah Tabrizi, Joint Head of the Department of Neurodegenerative Disease at UCL. Professor Tabrizi outlined the two-step pathogenesis of Huntington’s disease (HD): a rate driver involving somatic expansion of CAG repeats, and a toxicity driver arising from exon 1a fragment proteins and N-terminal proteolytic fragments of the mutant HTT gene, noting that most therapies to date have focused on the latter (Donaldson et al., 2026). She shared the development of AMT-130, a microRNA-based gene therapy targeting HTT, which has demonstrated early promising evidence for reductions in disease progression including patient-valued functional outcomes such as return to employment (Farag et al., 2025). She also highlighted work on MSH3 antisense oligonucleotide (ASO) treatment to slow somatic expansion in striatal-enriched human neurons, pointing to shared therapeutic opportunities across somatic expansion disorders (Bunting et al., 2025). Professor Tabrizi discussed future directions, including dosage optimisation, diversified therapeutic modalities, and plans for prevention studies, concluding with the remark: ‘we will get there’.

The session continued with Dr Maria Beatrice Panico, Chief Medical Officer at Scendea and former Medical Assessor at the MHRA, who shared extensive regulatory insights, emphasising the importance of balancing innovation with safety and oversight. She discussed routes of administration and their implications for blood–brain-barrier (BBB) bypass, on- versus off-target effects, and resulting safety and efficacy considerations. For example, only ~0.1% of intravenously administered IgG crosses the BBB, while AAV-based therapies face immunogenicity and liver exposure considerations, and nanoparticle approaches present challenges in long-term stability. Dr Panico highlighted regulatory complexities that can burden development, including approval of sites to perform invasive procedures for drug delivery, separate regulatory procedures for medical devices, and the responsibility of developers to provide sufficient evidence for novel products or components. She emphasised the importance of strategic patient selection in trial design and encouraged early regulatory engagement to mitigate and de-risk complex, resource-intensive development pathways.

## Panel discussion: *Whose Job Is It to Deliver?*

Paul Sharp and David Thomas (head of policy and public affairs at Alzheimer’s Disease Research UK), then joined the stage for a lively panel discussion, highlighting delivery as an applied science that extends far beyond the laboratory. The panel addressed practical feasibility in advanced therapeutic deployment including leveraging existing NHS infrastructure, collaborating with cancer biologists for viral manufacturing, and maximising translational resources. Challenges include: access to neurosurgical expertise, selection of appropriate disease models, biomarker development, UK-specific regulatory frameworks, reimbursement uncertainty, and constraints faced by small and medium-sized enterprises. Professor Tabrizi reflected on how recent high-profile trial failures have shifted attention towards the sustainability of drug delivery to the brain, particularly for gene therapies. The discussion also addressed workforce pressures and how emerging therapies are challenging established health economic frameworks, particularly those employed by the National Institute for Health and Care Excellence (NICE). Despite these challenges, the panel emphasised neurological therapeutics development as a ‘team sport’, requiring coordinated efforts across academia, industry, regulators, and healthcare systems. Dr Panico emphasised that it is never too early to seek regulatory advice and encouraged researchers to ‘start with the end in mind’. A recurring message from the panel was that successful delivery depends on early, coordinated decision-making rather than isolated scientific advances. Dr Panico closed the session by calling for the integration of disciplinary ‘languages’ to push boundaries and achieve more.

## Annual *BNA* awards ceremony

As per tradition, the *BNA* Festive Symposium featured an annual awards ceremony, promoting excellence in neuroscience at all career stages. The *BNA* Undergraduate Prize went to Jaya Sharma, who completed her BSc in Neuroscience at the University of Leeds. During her final year project, Jaya worked on artificial intelligence techniques to decode electroencephalogram data. The Postgraduate Prize went to Dr Ieva Andrulyte, whose PhD at the University of Liverpool explored lateralisation in brain connectivity, with a focus on language (Andrulyte et al., 2024). The Public Engagement Award was handed to Dr Susannah Walker on behalf of the exceptional Liverpool Neuroscience Group, for their inspiring ‘Bring Your Own Brain’ outreach programme within the BNA2025 International Festival of Neuroscience (Walker, 2025). Finally, the Outstanding Contribution to Neuroscience Award went to Professor Sarah Tabrizi, whose research into Huntington’s disease has paved the way for understanding and treating this and other devastating neurodegenerative conditions.

## What did early career researcher meeting reporters think of the symposium?

- Main takeaways from the symposium revolved around how emerging genetic and molecular insights are shaping therapeutic development. The fact that ALS/FTD-associated genes converge on shared mechanistic pathways provides a powerful basis for identifying tractable drug targets and accelerating the discovery process.
- It is clear from discussions on evolving therapeutic modalities and delivery technologies that the future belongs to targeted, patient-specific interventions.
- The symposium was notable for its well-structured programme, which integrated scientific, regulatory, and policy perspectives. The diversity of topics and speakers provided a comprehensive view of current challenges and opportunities.
- Networking opportunities during refreshment breaks were thoughtfully embedded, fostering interdisciplinary dialogue and collaboration.
- Importantly, the emphasis on future directions offered valuable guidance and motivation for early career researchers, situating their work within a broader landscape of innovation and patient impact.

## Conclusion

The *BNA* Festive Symposium 2025 explored challenges and opportunities of neuroscience-grounded therapy development. From gene therapy and imaging science to drug discovery and regulatory strategy, speakers traced the many steps involved in turning promising ideas into workable therapies. The symposium fostered a forward-looking but realistic openness, centring on patient-defined meaningful outcomes, and recognising central nervous system delivery as an interdisciplinary challenge. Neuroscience is not only about discovering biological targets, but also about making therapies deliverable within healthcare systems. Despite scientific progress and cross-sector collaboration, the symposium emphasised that the broader health system remains fragmented, slowing translational research and limiting equitable patient access. Neighbourhood care approaches were highlighted as essential for reaching deprived communities and ensuring that biomarker testing, risk profiling, and early‑intervention pathways do not reinforce inequalities. Overall, the symposium offered a panoramic survey of how new treatments for brain disorders are conceived, refined and ultimately delivered to patients.
